# The “Journal”

**Published:** 1859-12

**Authors:** 


### THE JOURNAL.

With the present number terminates the second year of its existence under its present name, the sixteenth of the entire series. During the nine months which we have had charge of it we have been cheered and encouraged by the approbation of its patrons and the courtesy of our friends of the medical press. Our list has increased; the medical journals throughout the entire country, with a single exception, have shown a friendly spirit. We enter upon a new year with hope and confidence.

The same general plan will be continued in its management. In order to add to its interest, however, a series of illustrated contributions to surgery by the senior editor will be given during the year. There is one point upon which a word of explanation may be due to some of our subscribers. We have at different times sent out printed slips to those who are in arrear. These seem to have been taken by those who have paid as addressed to themselves as well as to others.

This was not intended; and our reason for sending them to all was that so many cases were found to exist where subscriptions have been paid, or claimed to have been paid, but which the books did not show, that we adopted this course (borrowed from some of the best of our exchanges) as the surest method of communicating with those in arrear.

In order to guard against the recurrence of such an accident, and to remedy as far as possible the irregularities which have heretofore crept in, Prof. EPHRAIM INGALS will hereafter be associated with the senior editor as joint proprietor and editor. Dr. Ingalls will have charge of the books, circulation and exchanges, and communications may be addressed to him or to the senior editor.

